# Intraosseous Inflammatory Myofibroblastic Tumor in the Mandible: A Rare Pathologic Case Report

**DOI:** 10.1155/2014/565478

**Published:** 2014-09-01

**Authors:** Dale E. Stringer, Chad N. Allen, Katina Nguyen, Rahul Tandon

**Affiliations:** ^1^Loma Linda University, 11092 Anderson Street, 3rd Floor, Room 3307, Loma Linda, CA 92350, USA; ^2^Division of Oral and Maxillofacial Surgery, Parkland Memorial Hospital, University of Texas Southwestern Medical Center, 5323 Harry Hines Boulevard, Dallas, TX 75390-9159, USA

## Abstract

Inflammatory myofibroblastic tumor (IMT) is an extremely rare lesion found in the maxillofacial region. Its frequency diminishes further when found in the bone. Although classification has varied throughout its history, the histologic features are often diagnostic, particularly with its strong association with anaplastic lymphoma kinase-1 (ALK-1) staining. The current mode of treatment for such a lesion is surgical removal with careful followup. In this rare case report, we describe the diagnosis and treatment in a 16-year-old male. Although this rare pathology can present as—and at times mimic—more serious pathologies, it is important for the attending surgeon to initially manage the pathology conservatively.

## 1. Introduction

Inflammatory myofibroblastic tumor (IMT) is a very rare lesion and is even more rare in the maxillofacial region. First described in 1984, it underwent changes in its name for the next 10 years until the World Health Organization settled on the current terminology in 1994 [1–3]. It has been referred to in the literature by many other names, including xanthogranuloma, fibrous xanthoma, plasma cell granuloma, and inflammatory pseudotumor [4]. These tumors can arise from various anatomic locations, which can involve nearly every subspecialty in surgical oncology [2, 5]. Although more likely to be found in the lungs, extrapulmonary IMTs display more aggressive tendencies and can occur between 14 and 18% of the time [6, 7]. While these tumors are extremely rare, they are even more uncommon within bone [8, 9].

In the past, questions surrounding its origins have varied, with some describing it as a reactive process rather than a neoplasm [10]; oral IMTs have not, as of this moment, demonstrated any infectious etiologies [1]. When observed in the oral cavity, its aggressive nature may indicate a potentially malignant condition; however, the prognosis is usually not as dire [11, 12]. Diagnosis is usually confirmed histologically, which characterizes the lesion as a proliferation of fibroblasts and myofibroblasts mixed with plasma cells, eosinophils, and lymphocytes. In some cases, it has been described as resembling a sarcoma [13]. In this case report, we present a very rare case of a 16-year-old male who presented with an intraosseous inflammatory myofibroblastic tumor in the mandible.

## 2. Patient and Methods

**Figure 1:**
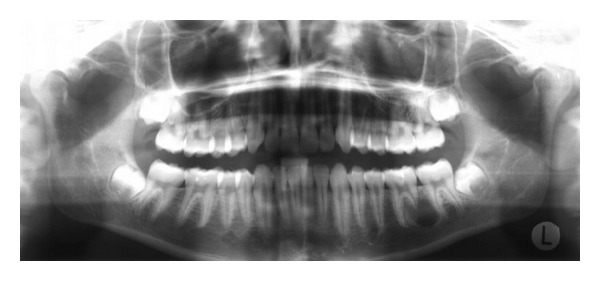
Initial panoramic radiograph.

**Figure 2:**
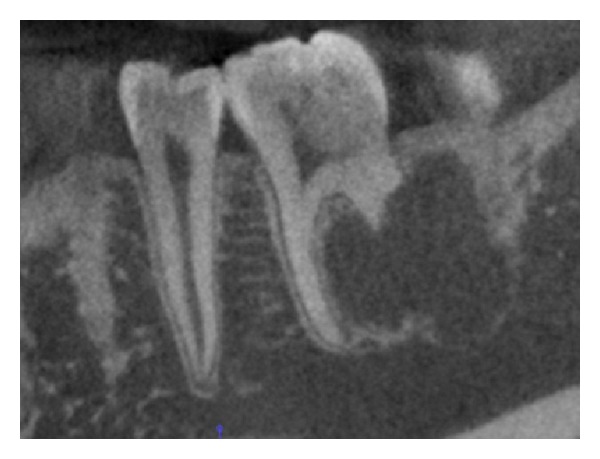
Panoramic radiograph demonstrating unilocular radiolucency with erosion of the distal root of tooth #19.

A 16-year-old boy was referred to our office after a routine radiographic examination revealed a radiolucent lesion in the left mandible (Figure 1). The lesion presented as an asymptomatic, intraosseous lesion in the left mandibular molar region as demonstrated by panoramic radiograph. Radiographic evaluation revealed a well-circumscribed radiolucent circular lesion obliterating the apical portion of the distal root of tooth #19; after nearly 3.5 months, the lesion had extended distally to include a small portion of the mesial root of tooth #18 (Figure 2). The preoperative diagnosis was consistent with a solid tumor.

### 2.1. Surgical Biopsy of Lesion

The patient was taken to the operating room and was subsequently induced into general anesthesia. A bovie electrocautery was then used to make a mucosal incision below the mucogingival junction adjacent to the first and second mandibular molars. A full-thickness mucoperiosteal flap was then reflected. A side-cutting bur was then used to remove a portion of the buccal cortical bone in the area of the distal root of tooth #19. After the cortex was removed, we noted that the bur easily “sunk” into the area of the lesion. No gross bleeding was encountered and the osteotomy was then widened to ensure better visualization. The lesion was then visually located and appeared solid and yellow in color. The lesion was removed in strands, easily separating from the surrounding bone. The entire lesion was curetted out, leaving an intact lingual cortex. The biopsied sample was then sent as a frozen section. The hospital general pathologist was not able to determine the definitive diagnosis of the lesion and recommended outside consultation. The cavity was irrigated with normal saline and the site was packed with Avitene and was closed primarily.

**Figure 3:**
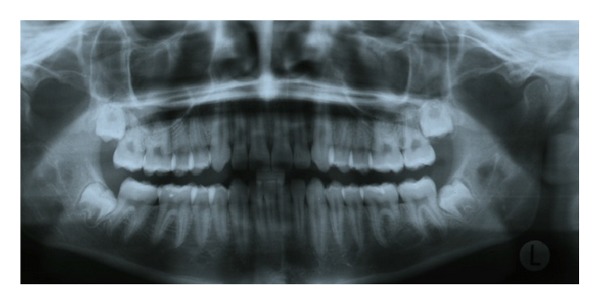
Six-month panoramic radiograph demonstrating no recurrence of lesion, with nascent bone formation.

The patient was recalled for a 6-month followup and demonstrated no clinical or radiologic evidence of recurrence. Postoperative panorex (Figure 3) demonstrates improved healing of the surgical site with evidence of new bone formation. The patient does not report any clinical symptoms or display any signs of recurrence of the lesion.

### 2.2. Pathologic Report

**Figure 4:**
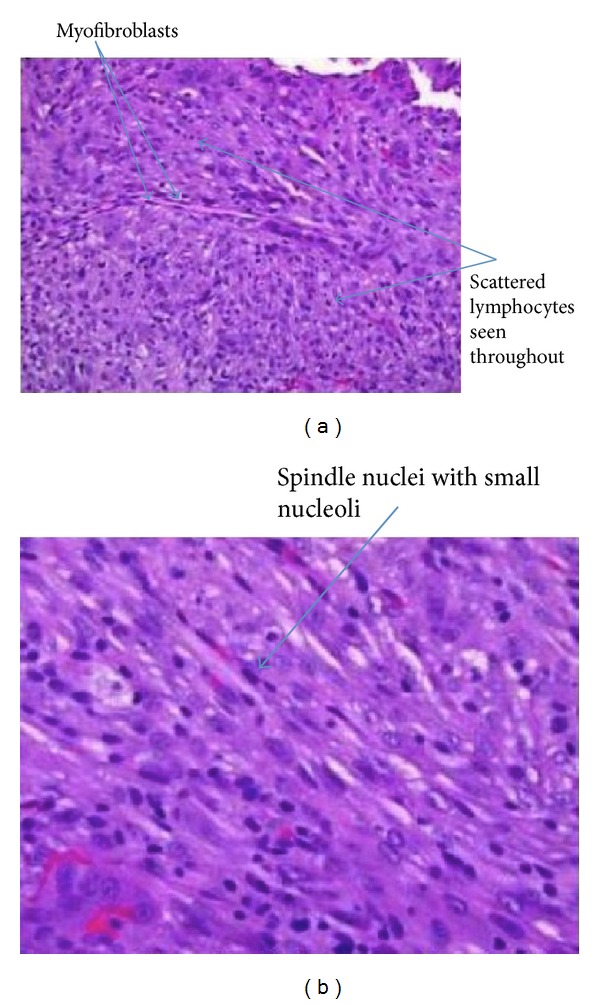
(a) Proliferation of plump spindle cells, with occasional giant cells and intermixed lymphocytes. Arrows illustrate lymphocytes and myofibroblasts. (b) Plump spindle cells with intermixed lymphocytes. Arrows demonstrating nuclei.

Sections of the submitted tissue, which were stained with hematoxylin and eosin, demonstrated plump spindle cells with an appearance suggestive of myofibroblasts, with scattered lymphocytes (Figure 4(a)). The spindle cell nuclei were plump, with small nucleoli, and lacked the cytologic features of malignancy (Figure 4(b)). Minimal—if any—extracellular matrix was produced by the lesional cells. The tumor cells showed moderate cytoplasmic staining for smooth muscle actin, but no staining for desmin. The lesional spindled cells showed diffuse and strong cytoplasmic staining for ALK-1 by immunohistochemistry. Anaplastic lymphoma kinase (ALK) is a novel receptor tyrosine kinase, possessing both a transmembrane domain and an extracellular domain. The ALK gene can be oncogenic in multiple ways and has shown expression in several other tumors [14].

The positive staining for ALK-1 strongly supports the diagnosis of inflammatory myofibroblastic tumor. Although not considered malignant and, therefore, not likely to metastasize, appropriate observation for any signs of recurrence must be undertaken.

## 3. Discussion

Although IMT can arise anywhere in the body, its occurrence in the head and neck region remains extremely limited. In fact, some authors state that there are only a handful of well-documented reports of IMT within the oral cavity in the literature [15]. While this dearth of cases can be attributed to its rarity in the oral cavity, another explanation could be that its classification was only unified recently [2]. This trend becomes even narrower once the lesion is found within bone. When presenting radiographically, it can resemble several neoplastic and reactive lesions, such as ameloblastic fibroodontoma and periapical granuloma. Additionally, histologically it can resemble any tumor or inflammatory process that also possesses myofibroblasts and fibroblasts. Nevertheless, the characteristics of the lesion, both clinically and histopathologically, demonstrate its unique identity.

Recently, lesions that demonstrate positive staining for ALK have shown to be a trademark feature of IMT, as seen in anywhere between 40% and 60% of the cases [16, 17]. Another possible link that has been theorized is the effects of postirradiation therapy; however this has yet to be fully established [12]. Demographically, the lesion exhibits a wide range; in several studies done on the intraoral variant, there was a mixture of males and females, with an age range between 19 and 77 years [18]. Our patient was relatively younger when compared with other studies; however this may be due to the relative infrequency of this pathology.

The treatment of choice in many instances, including ours, was simple surgical removal, with careful followup ensuring no recurrence. Although spontaneous disappearance with or without steroid therapy has been reported [19], we felt it was in the patient's best interest to continue with the well-established surgical excision. Intraosseous involvement can present challenges not commonly seen in the soft tissue variant, namely, anatomical considerations such as blood vessels and nerves. Radiographic imaging and computed tomography are currently the best methods for determining the amount of bone destruction and infiltration of the tumor.

## 4. Conclusion

IMT of the oral cavity, which is a rare entity on its own, is even more rare when found within the bone. Multiple pathologies that resemble IMT can only be excluded through a complete pathologic assessment via immunohistochemistry and other analyses. Although the behavior of IMT can appear malignant, it is important that the clinician properly determines the pathology before undertaking needless radical treatment. However, it is important that if presented with such a case the surgeon places the patient on regular follow-up visits to rule out recurrence.
